# Low Muscle Quality Predicts Creatinine-Based eGFR Overestimation Better than Skeletal Muscle Mass in Patients with Chronic Kidney Disease

**DOI:** 10.3390/biomedicines14102225

**Published:** 2026-10-01

**Authors:** Yukari Mae, Tomoaki Takata, Kensuke Kawata, Yudai Fujino, Sosuke Taniguchi, Shotaro Hoi, Takuji Iyama, Hajime Isomoto

**Affiliations:** Division of Gastroenterology and Nephrology, Faculty of Medicine, Tottori University, Yonago 683-8504, Tottori, Japan

**Keywords:** chronic kidney disease, phase angle, muscle quality, estimated glomerular filtration rate, skeletal muscle mass, bioelectrical impedance analysis, creatinine clearance

## Abstract

**Background/Objectives:** Accurate assessment of the glomerular filtration rate (GFR) is essential for managing chronic kidney disease (CKD). Although creatinine-based estimated GFR (eGFRcr) may be affected by reduced muscle mass, the contribution of muscle characteristics beyond muscle mass remains unclear. This study investigated whether phase angle (PhA), a bioelectrical impedance analysis-derived indicator associated with muscle quality, provides complementary information beyond appendicular skeletal muscle mass (ASM) regarding discordance between eGFRcr and a creatinine clearance (CCr)-based reference estimate. **Methods:** We enrolled 86 adults with CKD not receiving maintenance dialysis. CCr was calculated from 24 h urine collections, indexed to a standard body surface area (BSA) of 1.73 m^2^, and multiplied by 0.715. KOI was calculated as eGFRcr divided by (BSA-indexed CCr × 0.715). A KOI > 1.2 was used as an exploratory threshold for clinically significant discordance. **Results:** KOI > 1.2 was observed in 24.4% of patients. KOI was negatively correlated with ASM (r = −0.301, *p* = 0.005) and PhA (r = −0.451, *p* < 0.001). Adding PhA to a multivariable linear regression model including ASM improved model performance (adjusted R^2^, 0.060–0.200). Multivariable logistic regression, including ASM and PhA as explanatory variables, showed that only PhA remained independently associated with KOI > 1.2 (odds ratio, 0.258; 95% CI, 0.113–0.585; *p* = 0.001). **Conclusions:** PhA may provide complementary information beyond skeletal muscle mass for identifying patients with greater discordance between eGFRcr and corrected, BSA-indexed CCr. Further studies using measured GFR are needed to validate these findings.

## 1. Introduction

The accurate assessment of kidney function is fundamental to the diagnosis, staging, prognostic evaluation, and therapeutic management of chronic kidney disease (CKD) [1]. Although the glomerular filtration rate (GFR) can be measured directly using the clearance of exogenous filtration markers, such as inulin or iothalamate, these methods are labor-intensive, expensive, and impractical for routine clinical practice [2]. Consequently, estimated GFR (eGFR) derived from serum creatinine has become widely used for routine assessment of kidney function. Although the CKD–EPI equation is widely used internationally [3,4], population-specific equations have also been developed for the Japanese population [5].

Despite its widespread use, creatinine-based eGFR (eGFRcr) has inherent limitations because serum creatinine is influenced not only by glomerular filtration but also by endogenous creatinine generation [6]. Since creatinine is primarily produced by skeletal muscle, reduced muscle mass may cause an overestimation of eGFRcr independent of kidney function [7]. Patients with CKD are particularly susceptible to sarcopenia because aging-related muscle loss is further exacerbated by CKD-specific factors [8,9]. In addition to reduced muscle mass, muscle quality deteriorates through multiple interacting mechanisms, including uremic toxin accumulation, insulin resistance, chronic inflammation, oxidative stress, mitochondrial dysfunction, hormonal abnormalities, reduced physical activity, and nutritional abnormalities. These factors promote intramuscular fat accumulation and fibrosis, impair muscle regeneration, and disrupt the balance between muscle protein synthesis and degradation [10,11,12,13]. Therefore, the eGFRcr should be interpreted with caution in patients with impaired muscle health, including those with sarcopenia, frailty, and protein-energy wasting [14,15]. These limitations of creatinine-based equations have long been recognized, mostly from the perspective of muscle quantity, which represents only one aspect of skeletal muscle health.

Recently, muscle quality has gained attention as an essential component of skeletal muscle health. Current international consensus statements on sarcopenia recommend evaluating muscle quality as well as muscle mass, considering that deterioration in muscle quality may precede or occur independently of muscle loss and is closely associated with impaired physical performance and adverse clinical outcomes [15,16,17]. PhA reflects cellular integrity, cell membrane function, and body cell mass and has been used as a BIA-derived indicator associated with muscle quality [18]. In patients with CKD, lower PhA values have been consistently associated with malnutrition, sarcopenia, frailty, and poor clinical outcomes, including reduced quality of life and all-cause mortality [19,20,21]. Because PhA may reflect aspects of the structural and functional characteristics of skeletal muscle [22], it may capture aspects of endogenous creatinine-generating capacity that are not fully represented by skeletal muscle mass alone.

Nevertheless, the potential contribution of muscle quality to the interpretation of eGFRcr remains largely unexplored. Reduced skeletal muscle mass is a well-recognized source of error in eGFRcr [7,14,15]. However, it remains unknown whether BIA-derived indicators associated with muscle quality provide additional information beyond skeletal muscle mass. To the best of our knowledge, no previous study has investigated the association between PhA and discordance between eGFRcr and a CCr-based reference estimate. We hypothesized that PhA would be more strongly associated with this discordance than muscle quantity. Therefore, this study aimed to investigate whether PhA provides additional information beyond skeletal muscle mass in interpreting eGFRcr and identifying patients with greater discordance between eGFRcr and a CCr-based reference estimate.

## 2. Materials and Methods

### 2.1. Study Population and Design

This prospective observational study was conducted at the Department of Nephrology, Tottori University Hospital (Yonago, Japan) between April 2021 and June 2025. Patients with CKD who underwent bioelectrical impedance analysis (BIA) and 24 h urine measurement to evaluate creatinine clearance (CCr) during the study period were enrolled. Patients with an eGFR < 60 mL/min/1.73 m^2^ or a urinary protein-to-creatinine ratio (UPCR) > 0.15 g/gCr persisting for at least 3 months were considered to have CKD [23]. Patients were excluded if they were receiving maintenance dialysis, younger than 20 years, or had incomplete clinical or body composition data. The study was conducted in accordance with the principles of the Declaration of Helsinki and approved by the Institutional Review Board of Tottori University Hospital (Approval No. 20A186). Written informed consent was obtained from all participants before enrollment.

### 2.2. Body Composition Analysis

Body composition analysis was performed on the same day as blood sampling using BIA (MC-780A-N; TANITA, Tokyo, Japan). During the measurements, the participants stood barefoot on the footplate electrodes while holding the handgrip electrodes according to the manufacturer’s instructions. Height was measured using a stadiometer (Seca 228; Seca Nihon, Chiba, Japan) and body weight was measured using BIA. Body mass index (BMI) was calculated as body weight divided by height squared (kg/m^2^). The appendicular skeletal muscle mass (ASM) was measured using BIA. Muscle quality was assessed by PhA, which was calculated from resistance (R) and reactance (Xc) measured at 50 kHz according to the following equation: PhA (°) = arctangent (Xc/R) × (180/π). Blood pressure was measured in a seated position after at least 5 min of rest using an automated sphygmomanometer. Blood pressure values recorded during routine clinical practice were used in the present analyses.

### 2.3. Laboratory Data and Definitions

Blood and 24 h urine samples were collected on the study day as part of routine clinical assessment. Serum albumin, total protein, and creatinine concentrations were measured at the clinical laboratory of Tottori University Hospital. Serum and urinary creatinine concentrations were determined by enzymatic assays. Because the present study included only Japanese participants, eGFRcr was calculated using the Japanese Society of Nephrology equation, which was developed and validated for the Japanese population [5]. CCr was calculated from 24 h urine collections using the standard formula. BSA was calculated using the Mosteller formula: BSA (m^2^) = √[height (cm) × weight (kg)/3600]. CCr was then normalized to a standard BSA of 1.73 m^2^ using the following equation: indexed CCr = CCr × 1.73/BSA. Because CCr systematically overestimates measured GFR due to tubular creatinine secretion, a correction factor of 0.715 was applied to the BSA-indexed CCr to approximate measured GFR, based on the previously reported ratio of measured GFR to CCr [5]. To quantify the degree of eGFRcr overestimation relative to this corrected reference estimate, we defined a novel parameter, the Kidney function Overestimation Index (KOI), as follows: KOI = eGFRcr/(indexed CCr × 0.715). Higher KOI values indicated a greater degree of overestimation of kidney function by eGFRcr. A KOI > 1.2 was used as an exploratory threshold for clinically meaningful discordance between eGFRcr and the corrected, BSA-indexed CCr. This threshold was not intended to represent a validated clinical cutoff but was selected based on the concept described in the prior literature that a relative difference of approximately 20% between kidney function estimates may indicate clinically meaningful discordance [24].

### 2.4. Statistical Analysis

Continuous variables were presented as the mean ± standard deviation (SD) or median (interquartile range [IQR]), as appropriate according to their distribution. Categorical variables are expressed as frequencies and percentages. The normality of continuous variables was assessed using the Kolmogorov–Smirnov test. For descriptive analyses, participants were categorized into two groups according to the KOI: KOI ≤ 1.2 and KOI > 1.2. Comparisons between groups were performed using Student’s *t*-test or the Mann–Whitney U test for continuous variables, as appropriate, and the chi-square test for categorical variables. The association between the KOI and clinical variables was evaluated using Spearman’s rank correlation analysis. To address the potential influence of skeletal muscle mass on creatinine clearance, the correlation between BSA-indexed CCr and ASM was additionally assessed using Spearman’s rank correlation analysis. Multiple linear regression analysis was subsequently performed to identify factors independently associated with KOI as a continuous variable. A base model including age, sex, BMI, and ASM was constructed, after which PhA was added to evaluate its incremental explanatory value beyond skeletal muscle mass. Model performance was assessed using the adjusted coefficient of determination (adjusted R^2^) and Akaike’s information criterion (AIC). To explore potential interactions between PhA and other covariates, interaction terms between PhA and age, sex, BMI, and ASM were additionally examined. Multicollinearity was assessed using the variance inflation factor (VIF). Multivariate logistic regression analysis was performed to identify the factors independently associated with a clinically significant overestimation of kidney function (KOI > 1.2). Two logistic regression models were evaluated: Model 1 included age, sex, BMI, and ASM, whereas Model 2 additionally included PhA. The discriminative performance of the models was assessed using the area under the receiver operating characteristic curve (AUC), and the difference in AUCs between the two models was evaluated using DeLong’s test. Receiver operating characteristic (ROC) curve analysis was performed to evaluate the ability of PhA to discriminate patients with KOI > 1.2 from those with KOI ≤ 1.2. The optimal cut-off value was determined using the maximum Youden index. All statistical analyses were performed using StatFlex for Windows (version 6.0; Artec Co. Ltd., Osaka, Japan). All statistical tests were two-tailed, and a *p*-value < 0.05 was considered statistically significant.

## 3. Results

### 3.1. Patient Characteristics

In total, 102 patients were prospectively enrolled in this study. After applying predefined exclusion criteria, 86 patients were included in the final analysis. Baseline characteristics of the study population are summarized in Table 1. The study population comprised 54 men and 32 women, with a mean age of 58.9 ± 15.6 years. The median eGFRcr was 47.6 mL/min (IQR, 26.3–68.2), the median CCr was 63.2 mL/min (IQR, 25.3–88.5), and the median UPCR was 1.37 g/gCr (IQR, 0.44–3.23). According to the predefined definition of clinically significant overestimation, 65 patients (75.6%) were classified into the KOI ≤ 1.2 group, whereas 21 patients (24.4%) were classified into the KOI > 1.2 group. Compared with the KOI ≤ 1.2 group, patients with KOI > 1.2 had significantly lower body weight (57.0 [IQR, 46.2–74.7] vs. 67.4 [IQR, 57.7–76.2] kg, *p* = 0.047), ASM (18.8 ± 5.3 vs. 22.1 ± 5.1 kg, *p* = 0.013), PhA (4.30 ± 0.9° vs. 5.43 ± 1.0°, *p* < 0.001), eGFRcr (28.5 [IQR, 19.2–52.8] vs. 49.6 [IQR, 34.7–71.0] mL/min, *p* = 0.023), and CCr (24.8 [IQR, 15.0–47.7] vs. 70.0 [IQR, 46.8–101.4] mL/min, *p* < 0.001). No significant differences were observed between the two groups with respect to age, sex, height, BMI, systolic or diastolic blood pressure, serum albumin level, or UPCR.

### 3.2. Correlations Between KOI and Clinical Variables

In univariate correlation analyses, KOI was significantly negatively correlated with ASM (r = −0.301, *p* = 0.005) and PhA (r = −0.451, *p* < 0.001), whereas no significant correlations were observed with age (r = 0.186, *p* = 0.087) or BMI (r = −0.140, *p* = 0.199) (Table 2).

To further examine the association between PhA and KOI at higher KOI levels, we performed a stratified analysis using a KOI threshold of 1.2. Among patients with KOI > 1.2 (*n* = 21), PhA showed a negative but non-significant correlation with KOI (r = −0.317, *p* = 0.162), whereas no significant association was observed in patients with KOI ≤ 1.2. To address the potential influence of skeletal muscle mass on creatinine clearance, we additionally examined the correlation between BSA-indexed CCr and ASM. BSA-indexed CCr was not significantly correlated with ASM (r = 0.044, *p* = 0.686).

### 3.3. Multiple Linear Regression Analysis

To investigate whether PhA provides additional explanatory value beyond conventional indices, including ASM, multiple linear regression analysis was performed (Table 3). In Model 1, which included age, sex, BMI, and ASM as explanatory variables, only ASM was independently associated with KOI. In Model 2, in which PhA was added to Model 1, ASM and PhA remained independently associated with KOI. Furthermore, the adjusted R^2^ increased from 0.060 in Model 1 to 0.200 in Model 2, with a corresponding decrease in the AIC from 73.6 to 60.6, indicating that PhA may provide information that would not be captured by ASM alone.

To explore potential interactions between PhA and other covariates, interaction terms between PhA and age, sex, BMI, and ASM were additionally examined. No statistically significant interactions were observed between PhA and age (*p* = 0.570), sex (*p* = 0.757), or BMI (*p* = 0.211). A statistically significant PhA × ASM interaction was observed in the linear regression analysis (*p* = 0.036); however, the interaction term showed substantial multicollinearity (VIF = 52.4), and the corresponding interaction was not statistically significant in the logistic regression analysis for KOI > 1.2. Therefore, the PhA × ASM interaction was considered an exploratory finding and was not incorporated into the primary model.

### 3.4. Factors Independently Associated with KOI > 1.2

Multivariable logistic regression analysis was performed to identify factors independently associated with KOI > 1.2 (Table 4). In Model 1, which included age, sex, BMI, and ASM, none of the variables was independently associated with KOI > 1.2. In Model 2, after additionally including PhA, PhA was independently associated with KOI > 1.2 (odds ratio [OR], 0.258; 95% confidence interval [CI], 0.113–0.585; *p* = 0.001), indicating that each 1° decrease in PhA was associated with 3.88-fold higher odds of KOI > 1.2. The area under the receiver operating characteristic curve (AUC) was 0.699 (95% CI, 0.561–0.836) for Model 1 and 0.816 (95% CI, 0.711–0.918) for Model 2. The difference in AUCs was 0.117 and was statistically significant (DeLong’s test, *p* = 0.019).

### 3.5. Diagnostic Performance of PhA and ASM for Predicting KOI > 1.2

**Figure 1:**
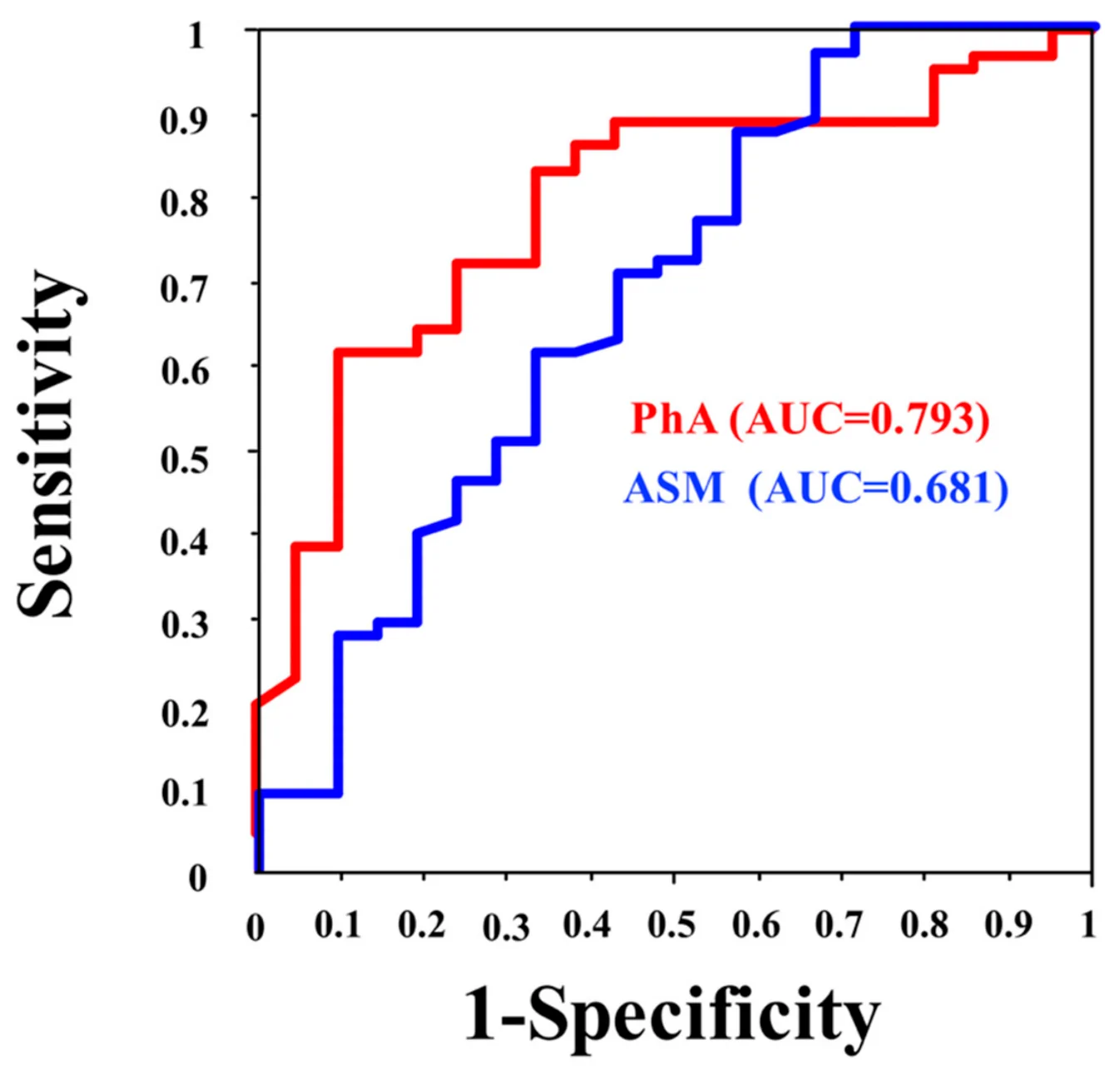
ROC curves of PhA and ASM for predicting KOI > 1.2. ROC curves were generated to evaluate the ability of PhA and ASM to discriminate patients with KOI > 1.2. The AUC was 0.793 for PhA and 0.681 for ASM. The optimal PhA cutoff determined using the Youden index was 4.56°, with a sensitivity of 83.1% and a specificity of 66.7%. ROC: receiver operating characteristic; AUC: area under the curve; PhA: phase angle; ASM: appendicular skeletal muscle mass; KOI: Kidney function Overestimation Index.

ROC analyses were performed to evaluate the diagnostic performance of PhA and ASM in predicting clinically significant kidney function overestimation (KOI > 1.2) (Figure 1). The areas under the curve (AUC) were 0.793 for PhA and 0.681 for ASM. The optimal cutoff value for PhA determined using the Youden index was 4.56°, yielding a sensitivity of 83.1% and a specificity of 66.7%.

## 4. Discussion

### 4.1. Phase Angle Provides Additional Information Beyond Muscle Mass in Relation to Kidney Function Estimate Discordance

The primary finding of this prospective study was that PhA, a BIA-derived indicator associated with muscle quality, was more strongly associated with the degree of discordance between eGFRcr and the corrected, BSA-indexed CCr than skeletal muscle mass in patients with CKD. Specifically, PhA exhibited the strongest negative correlation with the KOI among the evaluated clinical variables. Furthermore, the addition of the PhA to the conventional model, including the ASM, improved the explanatory performance of the model, increasing the adjusted R^2^ from 0.060 to 0.200. Finally, in multivariate logistic regression analysis adjusted for potential confounders, including age, sex, BMI, and ASM, only PhA remained independently associated with greater discordance, defined by an exploratory KOI threshold of >1.2. Overall, these findings support our hypothesis that PhA may provide independent and incremental information beyond skeletal muscle mass to identify patients with greater discordance between eGFRcr and the corrected, BSA-indexed CCr.

### 4.2. Potential Mechanisms Linking Skeletal Muscle Characteristics to Discordance in Creatinine-Based Kidney Function Estimates

Several mechanisms may explain why PhA showed a stronger association with discordance between eGFRcr and the corrected, BSA-indexed CCr than conventional muscle mass parameters in patients with CKD. First, PhA is calculated from the relationship between electrical resistance and reactance measured by BIA at a fixed frequency, and is considered a surrogate marker of cellular integrity, membrane function, and the distribution of intracellular and extracellular water. Previous studies have demonstrated the clinical relevance of PhA as a prognostic biomarker in various populations [18,25,26]. Unlike conventional indicators of muscle quantity such as ASM, PhA may provide complementary information related to cellular integrity and functional characteristics that are not captured by muscle mass alone [16,18]. Second, serum creatinine concentration is affected by endogenous creatinine production, with skeletal muscle representing the major source of creatinine generation [7,27]. The creatinine-generating capacity may be influenced by muscle cellular integrity and functional status, as well as by the amount of muscle mass, and may therefore affect the estimation of kidney function. Alterations in skeletal muscle function may decrease endogenous creatinine production, resulting in lower serum creatinine concentrations and potential overestimation of kidney function by creatinine-based eGFR in patients with impaired kidney function [7,27]. However, creatinine clearance may also be influenced by skeletal muscle mass because urinary creatinine excretion partly depends on endogenous creatinine production. Thus, the use of CCr as the reference for the KOI does not completely eliminate the potential influence of muscle mass on the assessment of kidney function. To address this concern, we additionally examined the association between BSA-indexed CCr and ASM. In the present cohort, no significant correlation was observed between BSA-indexed CCr and ASM (r = 0.044, *p* = 0.686). In patients with CKD, skeletal muscle abnormalities are caused by multiple pathological processes, including chronic inflammation, accumulation of uremic toxins, metabolic abnormalities, oxidative stress, and impaired mitochondrial function [28,29]. These factors may induce qualitative alterations in the muscle, including impaired cellular function and changes in muscle composition [28,29,30]. Importantly, qualitative changes in skeletal muscles may not always parallel measurable reductions in muscle mass, suggesting that muscle quantity alone may not fully represent the functional characteristics of the muscle [16,28,31]. These findings highlight the limitations of conventional muscle mass indices [16,28]. Recent evidence suggests that the creatinine production rate may reflect overall muscle status rather than muscle mass alone [32].

Consequently, patients with CKD may maintain an apparently preserved ASM despite qualitative alterations in skeletal muscle, which may be associated with differences in creatinine-generating capacity that are not fully explained by muscle quantity alone. In contrast, PhA may capture physiological or cellular characteristics associated with muscle deterioration that are not fully represented by conventional muscle mass measurements [18,26]. Our finding that the PhA remained independent after adjusting for ASM supports the concept that PhA may provide additional information beyond muscle quantity when interpreting creatinine-based kidney function.

Previous studies, including ours, have demonstrated the clinical relevance of PhA as a BIA-derived indicator associated with skeletal muscle health and its association with clinically important outcomes [19,20,21]. These findings support the potential role of PhA as a complementary measure for evaluating skeletal muscle status in patients with CKD, where qualitative alterations in muscles may not be fully captured by conventional quantitative assessments [16,17].

Interestingly, the serum albumin levels did not differ significantly between the groups in the present study. Serum albumin is associated with nutritional status, systemic inflammation, and fluid status, which are common underlying pathophysiologies of skeletal muscle deterioration [33,34]. PhA may provide additional information about skeletal muscle cellular health and integrity that is not reflected in serum albumin levels. Therefore, assessment of PhA may provide clinically relevant complementary information for identifying patients with greater discordance between eGFRcr and the corrected, BSA-indexed CCr despite apparently preserved muscle mass and conventional nutritional markers.

### 4.3. Clinical Implications

The eGFRcr is a standard marker for CKD staging, medication dose adjustment, and routine assessment of kidney function in clinical practice [3,4]. Our findings suggest that the assessment of PhA may provide clinically useful complementary information when interpreting eGFRcr. In the multivariable analysis adjusted for age, sex, BMI, and ASM, each 1° decrease in PhA was associated with a 3.88-fold higher odds of having a KOI > 1.2, an exploratory threshold representing > 20% discordance between eGFRcr and the corrected, BSA-indexed CCr [24]. This finding suggests that even relatively small reductions in the PhA may have clinically meaningful implications when interpreting kidney function in patients with CKD. Patients with low muscle mass and low renal function may be particularly susceptible to overestimation of kidney function based on serum creatinine, as observed in the present study (Table 1). Therefore, additional assessment of muscle characteristics may be of particular value in these populations.

Because PhA can be measured non-invasively without additional cost or patient burden, it can be readily incorporated into routine body composition assessments. In the present study, a PhA cut-off of 4.56° was associated with greater discordance between eGFRcr and the corrected, BSA-indexed CCr, defined using an exploratory KOI threshold of >1.2. Although this PhA cut-off requires external validation, a low PhA may help identify patients with a greater likelihood of discordance between eGFRcr and the corrected, BSA-indexed CCr. In such patients, a complementary assessment of cystatin C-based eGFR or careful interpretation of eGFRcr may be warranted [4]. However, cystatin C-based eGFR may also be influenced by non-GFR-related factors including malignancy and may not provide an optimal assessment of kidney function in certain clinical settings. Therefore, cystatin C-based eGFR should not be considered a definitive alternative in all patients.

Future studies should compare the diagnostic performance of PhA with established approaches, including cystatin C-based equations and measured GFR using exogenous filtration markers [2,3]. Prospective multicenter studies are required to determine whether longitudinal changes in PhA are associated with changes in the discordance between creatinine-based kidney function estimates and measured GFR over time. Such studies will help clarify whether the incorporation of PhA into routine clinical assessments can improve the interpretation of creatinine-based eGFR in patients with CKD.

### 4.4. Strengths and Limitations

The strength of this study lies in its prospective design and direct comparison of the respective contributions of skeletal muscle quantity and PhA, a BIA-derived indicator associated with muscle quality, to discordance between eGFRcr and the corrected, BSA-indexed CCr in patients with CKD. In addition, all participants underwent standardized BIA together with 24 h urine collection, allowing the calculation of the KOI based on corrected, BSA-indexed CCr rather than relying solely on eGFRcr. This approach enabled a comprehensive evaluation of the relationship between muscle characteristics and discordance between creatinine-based kidney function estimates in a clinically relevant setting.

This study had some limitations. First, this was a single-center study with a relatively small sample size (*n* = 86), and all participants were Japanese. The number of patients with KOI > 1.2 was also relatively small (*n* = 21), limiting the statistical power of the stratified analysis and our ability to determine whether the association between PhA and KOI persisted at higher KOI levels. The generalizability of our findings to broader and more diverse CKD populations, including those with different ethnic, geographic, lifestyle, and dietary backgrounds, therefore remains uncertain. Because this study was based on a single-center cohort without an independent external validation cohort, the reproducibility of the observed associations in other clinical settings cannot be determined from the present data. External validation using independent cohorts from other institutions and countries will be necessary to assess the generalizability and robustness of these findings. In addition, exploratory interaction analyses suggested a potential interaction between PhA and ASM in the linear regression analysis; however, this finding should be interpreted cautiously because the PhA × ASM interaction term showed substantial multicollinearity (VIF = 52.4) and the corresponding interaction was not statistically significant in the logistic regression analysis for KOI > 1.2. Therefore, the potential interaction between PhA and ASM remains uncertain and should be further evaluated in larger independent cohorts. Second, although the participants were enrolled prospectively, the present analysis was cross-sectional; therefore, a causal relationship between PhA and discordance between eGFRcr and the corrected, BSA-indexed CCr could not be established. Third, the approximated GFR was derived from 24 h creatinine clearance using a correction factor of 0.715 rather than being measured directly using exogenous filtration markers such as inulin, iohexol, or iothalamate clearance. Because both eGFRcr and CCr are creatinine-based measures, residual dependence on creatinine generation and muscle characteristics cannot be completely excluded. However, BSA-indexed CCr was not significantly correlated with ASM in the present cohort. The KOI should therefore be interpreted as an index of discordance between eGFRcr and the corrected, BSA-indexed CCr rather than a definitive measure of true GFR overestimation. Although measured GFR would provide a more rigorous reference standard, we used 24 h CCr as a clinically accessible measure applicable to routine clinical practice. Future studies using measured GFR are warranted to validate these findings. Finally, although patients receiving maintenance dialysis were excluded, residual confounding related to hydration status and body size could not be completely excluded. Although CCr was normalized to a standard BSA and BMI was included as a covariate in the multivariable analyses, these adjustments may not fully account for differences in body composition and fluid distribution that could affect BIA-derived measurements. Dietary protein intake and other factors affecting creatinine metabolism could also not be completely excluded.

## 5. Conclusions

In conclusion, this study suggests that PhA may serve as a clinically useful complementary indicator for identifying patients with greater discordance between eGFRcr and the corrected, BSA-indexed CCr beyond the information provided by skeletal muscle mass alone. Therefore, the assessment of PhA may improve the interpretation of eGFRcr in patients with CKD. Further large-scale multicenter prospective studies using measured GFR are warranted to validate these findings and establish the clinical utility of incorporating PhA in the routine assessment of kidney function.

## Figures and Tables

**Table 1:** Baseline characteristics of the study participants according to the Kidney function Overestimation Index (KOI).

Parameter	Total (*n* = 86)	KOI ≤ 1.2 (*n* = 65)	KOI > 1.2 (*n* = 21)	*p* Value
Age (years)	58.9 ± 15.6	57.0 ± 15.3	64.6 ± 15.5	0.054
Sex (male/female)	54/32	43/22	11/10	0.256
Cause of renal disease				0.231
Glomerulonephritis	57.0%	60.0%	47.6%	
Diabetic nephropathy	15.1%	15.4%	14.3%	
Nephrosclerosis	9.3%	10.8%	4.8%	
Other/unknown	18.6%	13.8%	33.3%	
Height (m)	1.66 (1.60–1.71)	1.67 (1.61–1.71)	1.63 (1.52–1.70)	0.148
Body weight (kg)	63.8 (55.6–76.1)	67.4 (57.7–76.2)	57.0 (46.2–74.7)	0.047
BMI (kg/m^2^)	24.0 ± 4.2	24.5 ± 4.0	22.6 ± 4.6	0.085
sBP (mmHg)	133.0 (119.0–148.0)	130.0 (118.8–142.3)	147.5 (133.0–161.8)	0.085
dBP (mmHg)	79.0 (71.0–87.0)	80.0 (70.8–87.0)	79.0 (71.5–89.0)	0.880
ASM (kg)	21.3 ± 5.3	22.1 ± 5.1	18.8 ± 5.3	0.013
PhA (°)	5.15 ± 1.1	5.43 ± 1.0	4.30 ± 0.9	<0.001
Alb (g/dL)	3.55 (3.00–4.47)	3.70 (3.20–4.58)	3.30 (3.00–4.10)	0.183
eGFRcr (mL/min/1.73 m^2^)	47.6 (26.3–68.2)	49.6 (34.7–71.0)	28.5 (19.2–52.8)	0.023
CCr (mL/min)	63.2 (25.3–88.5)	70.0 (46.8–101.4)	24.8 (15.0–47.7)	<0.001
UPCR (g/gCr)	1.37 (0.44–3.23)	1.04 (0.33–2.97)	2.08 (1.09–4.56)	0.838

BMI, body mass index; sBP, systolic blood pressure; dBP, diastolic blood pressure; ASM, appendicular skeletal muscle mass; PhA, phase angle; Alb, serum albumin; eGFRcr, estimated glomerular filtration rate; CCr, creatinine clearance; UPCR, urine protein-to-creatinine ratio; KOI: Kidney function Overestimation Index. Values are presented as mean (standard deviation) for normally distributed variables, median [interquartile range] for non-normally distributed variables, or number (%) as appropriate. Group comparisons were performed using Student’s *t* test or the Mann–Whitney U test for continuous variables and the χ^2^ test for categorical variables, as appropriate.

**Table 2:** Spearman’s correlation between KOI and clinical variables.

Variable	Spearman’s r	*p* Value
Age	0.186	0.087
BMI	−0.140	0.199
ASM	−0.301	0.005
PhA	−0.451	<0.001

KOI, kidney function overestimation index; BMI, body mass index; ASM, appendicular skeletal muscle mass; PhA, phase angle. Spearman’s rank correlation coefficients (r) were used to evaluate the association between KOI and clinical variables.

**Table 3:** Multiple linear regression analysis for factors associated with KOI.

Variable	Model 1		Model 2	
	Stdβ	*p* Value	Stdβ	*p* Value
Age	0.109	0.341	−0.097	0.411
Sex (Ref. M)	0.063	0.670	0.257	0.078
BMI	0.053	0.681	0.184	0.138
ASM	−0.336	0.0499	−0.264	0.096
PhA	−	−	−0.559	<0.001

KOI, kidney function overestimation index; BMI, body mass index; ASM, appendicular skeletal muscle mass; PhA, phase angle. Model 1 included age, sex, BMI, and ASM. Model 2 included age, sex, BMI, ASM, and PhA. The adjusted R^2^ increased from 0.060 (Model 1) to 0.200 (Model 2).

**Table 4:** Multivariable logistic regression analysis for factors associated with KOI > 1.2.

Variable	Model 1		Model 2	
	OR (95% CI)	*p* Value	OR (95% CI)	*p* Value
Age	1.021 (0.984–1.059)	0.273	0.987 (0.944–1.032)	0.557
Sex (Ref. M)	0.677 (0.164–2.800)	0.590	0.195 (0.032–1.195)	0.077
BMI	0.984 (0.847–1.143)	0.833	1.075 (0.905–1.278)	0.401
ASM	0.866 (0.728–1.032)	0.108	0.879 (0.741–1.043)	0.141
PhA	−	−	0.258 (0.113–0.585)	0.001

OR, odds ratio; CI, confidence interval; Ref, reference; M, male; BMI, body mass index; ASM, appendicular skeletal muscle mass; PhA, phase angle; KOI, Kidney function Overestimation Index. Multivariable logistic regression analysis was performed to identify factors independently associated with a KOI score > 1.2. Odds ratios (ORs) and 95% confidence intervals (CIs) were determined. Model 1 included age, sex, BMI, and ASM, whereas Model 2 additionally included PhA. Variables were included in the models based on clinical relevance, and multicollinearity was assessed before model construction. The AUC was 0.699 (95% CI, 0.561–0.836) for Model 1 and 0.816 (95% CI, 0.711–0.918) for Model 2. The difference in AUCs between Model 2 and Model 1 was 0.117 (DeLong’s test, *p* = 0.019).

## Data Availability

The data presented in this study are not publicly available due to ethical and privacy restrictions, but they are available from the corresponding author upon reasonable request.

## Abbreviations

ASM	Appendicular skeletal muscle mass
AUC	Area under the curve
BIA	Bioelectrical impedance analysis
BMI	Body mass index
CCr	Creatinine clearance
CKD	Chronic kidney disease
CKD–EPI	Chronic Kidney Disease Epidemiology Collaboration
eGFR	Estimated glomerular filtration rate
eGFRcr	Creatinine-based estimated glomerular filtration rate
GFR	Glomerular filtration rate
IQR	Interquartile range
KOI	Kidney function Overestimation Index
OR	Odds ratio
PhA	Phase angle
ROC	Receiver operating characteristic
SD	Standard deviation
UPCR	Urine protein-to-creatinine ratio
